# Supplementary material for: Netcombin: An algorithm for constructing optimal phylogenetic network from rooted triplets
Source: PLoS One. 2020 Sep 18;15(9):e0227842. doi: 10.1371/journal.pone.0227842 (PMC7500971; doi:10.1371/journal.pone.0227842)
Supplement: S4 File — (ZIP) [file pone.0227842.s004.zip › SequenceData-Triplet/sequenceData-10-20-30-0-Triplet/length 200, numseq 40, rate 10^-7/4 seq.pdf]

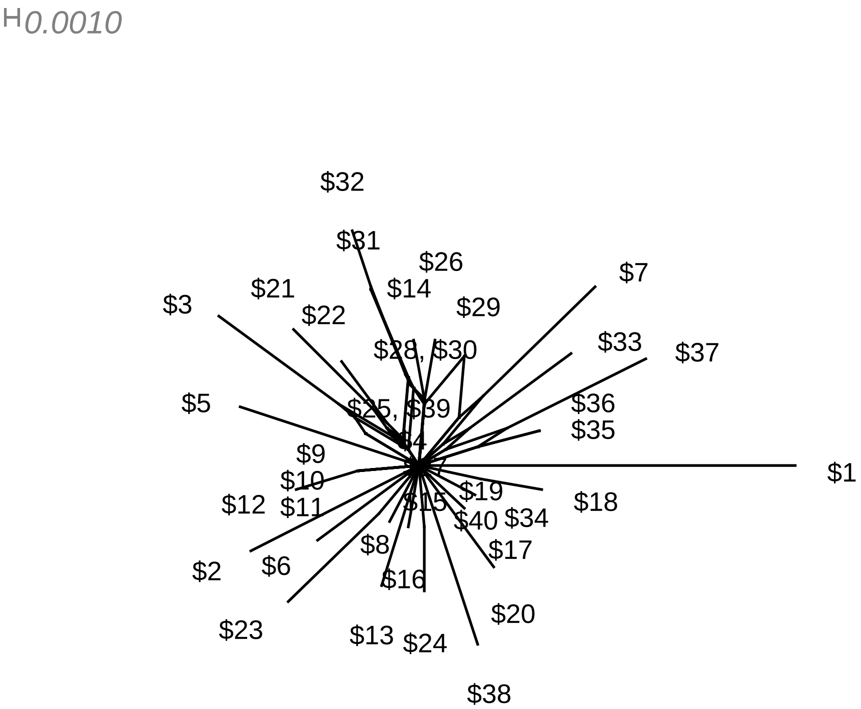
0.0010
$32
$31
$26
$7
$14
$21
$3
$22
$29
$33
$37
$28, $30
$5
$25, $39
$36
$35
$4
$9
$1
$10
$19
$12
$18
$11
$15
$34
$40
$8
$17
$2
$6
$16
$20
$23
$13
$24
$38
